# Wide Awake Local Anesthesia No Tourniquet Surgery of Carpal Tunnel Syndrome: Patients’ Experience and Recall Bias in a Day-Care Setting

**DOI:** 10.3390/medicina59050979

**Published:** 2023-05-19

**Authors:** Saulius Knystautas, Kęstutis Braziulis, Ernest Zacharevskij, Karolis Varkalys, Violeta Šimatonienė, Loreta Pilipaitytė

**Affiliations:** 1 Faculty of Medicine, Lithuanian University of Health Sciences, A. Mickevičiaus g. 9, LT-44307 Kaunas, Lithuania; 2 Department of Plastic and Reconstructive Surgery, Lithuanian University of Health Sciences, A. Mickevičiaus g. 9, LT-44307 Kaunas, Lithuania; 3 Department of Physics, Mathematics and Biophysics, Lithuanian University of Health Sciences, A. Mickevičiaus g. 9, LT-44307 Kaunas, Lithuania

**Keywords:** Wide-Awake Local Anesthesia No Tourniquet, patient satisfaction, recall bias, Carpal Tunnel Syndrome

## Abstract

*Background and Objective*: Wide-Awake Local Anesthesia No Tourniquet (WALANT) is a technique of local anesthesia commonly used in the surgical treatment of a wide variety of conditions affecting the upper extremity, including Carpal Tunnel Syndrome (CTS). The recent retrospective studies investigated patient experiences in a wide variety of hand disorder-related cases. The aim of our study is to evaluate patient satisfaction regarding open surgical treatment for CTS using the WALANT technique. *Material and Methods*: we enrolled 82 patients with CTS without medical record of surgical treatment for CTS. For WALANT, a hand surgeon used a combination of 1:200,000 epinephrine, 1% lidocaine, and 1 mL 8.4% sodium bicarbonate solution without tourniquet application and sedating the patient. All patients were treated in a day-care setting. For assessment of patient experience, Lalonde’s questionnaire was adapted. Participants completed survey twice: one month and six months after the surgical treatment was performed. *Results*: the median pre-operative pain score for all patients was 4 (range 0–8) after one month and 3 (range 1–8) after six months. The median intraoperative pain score for all patients was 1 (range 0–8) after one month and 1 (range 1–7) after six months. The median post-operative pain score for all patients was 3 (range 0–9) after one month and 1 (range 0–8) after six months. More than half (61% after one month and 73% after six months) of the patients responded by stating that their real experience of WALANT was better than their initial expectations. An absolute majority of patients (95% after one month and 90% after six months) would recommend WALANT treatment to their relatives. *Conclusions*: overall, patient satisfaction with treatment for CTS using WALANT is high. Furthermore, complications related to the performed treatment and persistent post-operative pain could be associated with more reliable patient recall of this healthcare intervention. A longer period of time between intervention and assessment of patient experience could possibly be a reason for recall bias.

## 1. Introduction

Carpal Tunnel Syndrome is the most prevalent entrapment neuropathy worldwide and causes numbness, pain, and tingling sensations in the hand and arm [1,2]. The individual risk factors of CTS are as follows: age, sex, diabetes, hypothyroidism, obesity, complications of systematic diseases, tobacco, and injury [3,4]. Recent literature indicated that increased pressure in the carpal tunnel, median nerve microcirculation lesion, median nerve connective tissue compression, and synovial tissue hypertrophy are the main interacting pathophysiologic mechanisms in the development of CTS [5]. Surgical treatment of CTS requires division of the transverse carpal ligament, which can be performed using endoscopic or open surgical methods. Moreover, the debate between proponents of open and endoscopic carpal tunnel surgery continues because both methods are well tolerated with no differences in functional outcomes, symptom severity, or complications [6,7]. Wide-Awake Local Anesthesia No Tourniquet is an innovative method of local anesthesia commonly used in surgical treatment of patients with CTS [8,9]. Dr. Donald H. Lalonde first described this method for the purpose of treating patients with upper extremity disorders in a day-care setting [10,11]. The technique is performed by a hand surgeon using a combination of epinephrine and lidocaine solution, which is injected into a surgical field for a conscious patient without tourniquet application. This method of local anesthesia obtains success in hand surgeon practice due to a possibility of achieving a bloodless surgical field without the discomfort of tourniquet application [12]. Evaluation of patient active hand function during the intraoperative period and reduction in the costs, risks, and inconveniences of sedation and general anesthesia are other advantages of using this local anesthesia method [13,14]. Furthermore, the WALANT method proved that it has similar patient satisfaction rates and analgesic effects when compared with regional anesthesia and tourniquet [15]. Contraindications for this method of local anesthesia are associated with peripheral ischemic disease or certain vasculopathies, including Raynaud’s phenomenon, Buerger disease, vasculitis, and scleroderma. Other conditions that would exclude a patient from WALANT include allergies or hypersensitivities to lidocaine or epinephrine [16,17]. A recent study compared WALANT with local anesthesia and tourniquet application. Tourniquets were the main cause of discomfort during surgeries. The WALANT technique was associated with better patient comfort during this surgery [12]. Patient involvement in healthcare decision-making is increasingly conceptualized as a fundamental measurement of healthcare quality. The literature suggests that evaluation of patient experience of performed treatment is an appropriate complement to clinical quality measures in healthcare. Furthermore, evaluation of patient experience may help assess the degree to which healthcare is patient centered [18,19,20]. In low- and middle-income countries, it is common to frame statements positively in patient satisfaction and experience surveys. Positive responses may reflect either bias induced via the positive framing or true satisfaction with the perceived treatment [21]. The primary aim of this study is to evaluate patient satisfaction with open surgical treatment of CTS using WALANT technique in the day-care setting of a single center of plastic and reconstructive surgery in Lithuania. The secondary aim of this study is to evaluate possible causes of recall bias in Dr. Lalonde’s WALANT patient satisfaction and experience questionnaire through comparing the answers given by the same CTS patient group’s members one and six months after surgical treatment. The majority of previous studies evaluated patient experience with WALANT in cases related to a wide variety of hand disorders [22,23]. To the best of our knowledge, this paper is the first to study the possible key issues causing recall bias in Dr. Lalonde’s WALANT patient satisfaction and experience questionnaire.

## 2. Materials and Methods

A retrospective study was conducted on patients with Carpal Tunnel Syndrome. Ethical approval for this study was obtained from Kaunas Regional Biomedical Research Ethics Committee (21 April 2022, No. BE-2-48). Patients were only included if they met all of the study’s inclusion criteria: they were older than 18 years old during inclusion in the study, diagnosed with CTS without a medical record of surgical treatment, and signed an informed consent form for participation in the research. Exclusion criteria excluded the following individuals: pregnant patients; patients with a history of systemic connective tissue diseases, diabetes mellitus, and ischemic heart disease; and patients with a known allergy to lidocaine or epinephrine. All patients were treated in the plastic and reconstructive surgery department of Kaunas clinics in a day-care setting. Surgeries were performed by two senior hand surgeons using the same surgical technique. For wide awake local anesthesia, surgeons used a combination of 1:200,000 epinephrine, 1% lidocaine, and 1 mL of 8.4% sodium bicarbonate solution without tourniquet application. Moreover, surgical field anesthesia in carpal tunnel release was achieved with 10–20 mL of combined solution. With permission, we adapted Lalonde’s questionnaire to evaluate participant satisfaction and experience of Wide-Awake Local Anesthesia No Tourniquet surgery. The survey consisted of two parts. The first part contained numerical rating scale (NRS)-type questions to assess pre-operative, intraoperative, and post-operative pain and anxiety levels. Scales were scored from 0–10 (0 meaning no pain or anxiety and 10 meaning the worst pain or anxiety imaginable). The second part of the survey contained questions related to overall patient experience and satisfaction before, during, and after the surgical treatment was performed. Participants completed the survey twice: one month and six months after the surgical treatment was performed. We ensured that the same participant completed the survey twice due to the opportunity to assess recall bias of patient. Furthermore, we decided to evaluate possible causes that may influence recall bias in patients through adding more direct questions about patients’ hand function during their follow-up visits six months after the treatment. Results after one month are considered a short-term outcome, while results after six months are considered a long-term outcome. The data were analyzed using Statistical Package for Social Sciences (SPSS). Pain and anxiety scores were not normally distributed; thus, they were presented as medians and intervals of values. The Wilcoxon test was applied for comparison of results after one and six months. Distribution of answers to the questions was presented in counts and percentages. The comparison of answers after one and six months was made using the McNemar and McNemar–Bowker tests. For hypothesis testing, a significance level of *p* < 0.05 was chosen.

## 3. Results

Eighty-two patients participated in our study. Approximately one quarter (26.8%) of all patients were male and remaining approximately three quarters (73.2%) were female; the mean age was 56 years old (range 39–89). All patients were treated in a day-care setting.

### 3.1. Experience of Pain and Anxiety

The median pre-operative pain score (during infiltration of local anesthetic) for all patients was 4 (range 0–8) after one month and 3 (range 1–8) after six months. The median intraoperative pain score (after local anesthesia was performed) for all patients was 1 (range 0–8) after one month and 1 (range 1–7) after six months. The median post-operative pain score (when effect of local anesthesia disappeared) for all patients was 3 (range 0–9) after one month and 1 (range 0–8) after six months (Table 1).

Patients were asked to compare their pain experience during surgery using the WALANT method with dental treatment procedures. An absolute majority of patients (93% after one and six months) answered that WALANT was less painful or there was no difference compared with dental treatment after one and six months. Approximately two-thirds (70% after one month and 61% after six months) of all patients responded that they took post-operative pain treatment after the surgery was performed. The difference was statistically significant regarding post-operative pain treatment comparing one- and six-month results (*p* < 0.05). Furthermore, one-third of all patients did not take post-operative pain treatment at all. An absolute majority of patients (97% after one month and 96% after six months) responded that post-operative pain treatment with drugs was successful. Approximately half (49% after one month and 59% after six months) of all patients responded that the first signs of post-operative pain were noticed during three to six hours following the surgery (Table 2). The median pre- and intra-operative anxiety scores for all patients were 2 (range 0–10) after one month and 1 (range 0–10) after six months. Post-operatively, patients experienced nearly no anxiety at all (Table 1).

### 3.2. Satisfaction during WALANT

Patients were asked to compare their expectations regarding CTS surgery using the WALANT method with their actual experience. More than half (61%) of all patients responded that their real experience of WALANT was better than their initial expectations one month after surgical treatment was performed. Approximately one-third (34%) of all patients responded that their real experience during WALANT matched their expectations one month after surgical treatment was performed. The difference in results between the real experience of WALANT and expectations was statistically significant both one and six months after surgical treatment was performed (*p* < 0.05) (Table 2). An absolute majority of patients (95% after one month and 90% after six months) would recommend WALANT treatment to their relatives. Respondents agreed (98% after one month and 93% after six months) that WALANT would be their method of choice for another hand surgery in the future, if required. Overall, WALANT was successful for all 82 patients. There was no need for regional or general anesthesia. None of the patients experienced complications associated with lidocaine toxicity after WALANT, such as headaches, dizziness, nausea, or vomiting. No cases required phentolamine for reversal.

### 3.3. Persistent Pain and Complications Relation with Surgery Experience

To evaluate the possible causal factors that could affect how patients recall their surgery experience, three additional questions were added to the questionnaire during follow-up after 6 months. Roughly one-fifth (17%) of all patients suffered persistent hand pain six months after surgery. Four patients (5%) responded that they had minor complications related to surgical treatment that did not require surgical intervention six months after the initial surgery was performed. Sixty-six respondents (81%) were satisfied with the outcome of the surgery (Table 2). Patients with persistent hand pain recalled intra- and post-operative pain, and their pre-, intra-, and post-operative anxiety scores were similar when comparing the results of the survey after one and six months. Moreover, patients with post-operative complications recalled that their pre-, intra-, and post-operative pain and anxiety scores were similar when comparing results after one and six months. Overall, patients with successful surgical treatment had significantly lower pain and anxiety scores six months after surgery. (Table 3).

## 4. Discussion

The WALANT technique was proven to be successful for common procedures, including surgical treatment for CTS. In a recent paper, a literature review was conducted with the purpose of evaluating the advantages and disadvantages of WALANT [16]. According to research data, WALANT was proved to be successful in a wide range of procedures. The main advantages of this method were intraoperative assessment with active participation of patient, downsizing of costs, and high patient satisfaction rates. A contemporary study analyzed patients’ pain and anxiety levels pre- (during infiltration), intra-, and post-operatively [24]. In this research, a broad spectrum of trauma patients were included. We chose to include only day-care setting patients with a CTS diagnosis, with the purpose of obtaining a homogenous sample of patients in distribution by age, gender, and diagnosis. Based on their statistical data, the mean anxiety score (1–10) was 4.0 pre-operatively, 2.5 intraoperatively, and 0.7 post-operatively. The mean pain score (1–10) was 3.8 during infiltration, 0.8 intraoperatively, and 0.4 post-operatively.

That study’s results regarding pre- and intra-operative pain evaluation are similar to our findings. However, pre-operative anxiety levels were significantly higher. We made a hypothesis that proposed that day-care setting surgery is less stressful because patients usually have time to prepare for upcoming surgery. In the case of trauma, patients experience much more stress because surgical treatment is unexpected. Similar to our results, 80% of their patients described WALANT as similar to or less painful than dental treatment. Moreover, 95% of patients responded that their overall experience was better than expected. This result is significantly higher compared with the results of our study (61% after one month and 73% after six months). Inequality in results can be explained through different levels of satisfaction among patients. In the case of trauma patients, the main objective is to restore integrity and essential function of the hand. After surgical treatment of trauma, the patients’ hand integrity and capability to perform essential functions are mostly restored within a couple months. Patients with CTS have only partial disruption of hand function, while manipulations of essential hand movements in daily activities are restricted only to a certain degree.

In a previously mentioned study [24] and in our research, more than 90% of patients would definitely recommend WALANT to relatives, which leads to assumption that overall satisfaction with WALANT is at a high level. In their research, approximately half of all patients responded that first signs of post-operative pain were noticed in the period three to six hours post-operation, which is a similar result to our study.

In our research, we recorded complications related to surgical treatment during an assessment conducted six months after the treatment was performed. Based on the data from our survey, four patients had minor complications related to surgical treatment that did not necessitate surgical intervention. Patients considered persistent tingling, numbness, and loss of strength in hand as complications related to surgical treatment. None of our patients suffered from post-operative infections.

In a contemporary article, a retrospective study was conducted to evaluate post-operative complications and infection rates in a group of patients undergoing CTS surgical treatment [25]. Similar to our findings, there were no infections recorded after surgical treatment of CTS with the WALANT method. Furthermore, rates of complications were compared between WALANT and Monitored Anesthetic Care (MAC). There was no difference in complication rates between the WALANT and MAC methods. Surgical treatment for CTS using the WALANT method can be safely performed without concern for any increased risk of infections or complications.

Currently, there is no consensus on the association between patient experiences and clinical outcomes. Some studies demonstrate that lower rates of complications and mortality are related to a better patient experience [26,27]. However, recent papers argue that there is no association between overall patient experience and clinical outcomes [28]. A cross-sectional study was conducted to evaluate associations between patient experiences and clinical outcomes. According to the study’s results, patient experience appears to be a separate quality measure that does not necessarily reflect good clinical outcomes of treatment [29]. Furthermore, patient-reported experience surveys can reflect patients’ pre-determined health care expectations and not their actual care experience. In our study, we found that complications related to surgical treatment and persistent post-operative pain could be associated with more reliable patient recall of healthcare interventions. Moreover, successful treatment and a long period of time from intervention to assessment of patient experience could possibly be reasons for recall bias. Patient-reported experience surveys have gained worldwide recognition as an indicator of health care quality. Despite this benefit, recall bias affecting patients’ experience and different patients’ expectations regardijg healthcare intervention remain the main limitations of patient-reported experience surveys for full-scale adaptability in healthcare quality analysis. We support the idea that it is important to be aware of the validity and reliability already undertaken for the patient-reported experience surveys. Furthermore, authors should undertake more robust testing of patient-reported experience surveys before utilizing them in their research.

In our study, we only involved patients with a diagnosis of CTS. For future studies, we recommend including a broader spectrum of diseases (such as Trigger finger or Dupuytren’s contracture) that are treated in a day-care setting. Furthermore, we recommend comparing patient satisfaction with the WALANT procedure with other methods of regional anesthesia. Moreover, further prospective randomized studies evaluating patient experience during WALANT are required to confirm our findings.

## 5. Conclusions

The findings of this study should be interpreted with caution and verified through further research; however, they indicate that WALANT does not seem to cause significant pain or stress for patients with CTS. The patients in this study expressed satisfaction with their pre-, intra-, and post-operative care in a day-care setting. Moreover, complications related to the treatment and persistent post-operative pain could be linked to more accurate patient recall of healthcare intervention. A long interval between intervention and assessment of patient experience could potentially be a reason for recall bias. Patient-reported experience surveys are emerging as one of the main indicators of healthcare quality; however, the relationship between patient experiences and clinical outcomes remains unclear. Our study supports the claim that WALANT is a safe, effective, and well-accepted method for hand surgery. Further prospective randomized studies are needed to confirm our findings.

## Figures and Tables

**Table 1:** Patient Numerical Rating Scale (NRS) pain and anxiety evaluation scores after 1 and 6 months.

Question	Evaluation Scores Median (Range)		*p* Value
	After 1 Month	After 6 Months	
Experience of pain during local anesthetic infiltration	4 (0–8)	3 (1–8)	<0.001
Experience of pain during surgery	1 (0–8)	1 (0–7)	0.004
Experience of pain after surgery	3 (0–9)	1 (0–8)	<0.001
Experience of anxiety before surgery	2 (0–10)	1 (0–10)	<0.001
Experience of anxiety during surgery	2 (0–10)	1 (0–10)	<0.001
Experience of anxiety after surgery	0 (0–8)	0 (0–8)	0.194

**Table 2:** Responses from patients with Carpal Tunnel Syndrome after 1 and 6 months.

Question	Distribution of Answers (Count, Percentage)		*p*
	After 1 Month	After 6 Months	
Please compare sensation of pain in wide awake hand surgery with dental procedures that caused pain			0.381
Wide awake hand surgery is less painful	56 (68.3)	60 (73.2)	
Wide awake hand surgery is more painful	4 (4.9)	4 (4.9)	
There is no difference	20 (24.4)	16 (19.5)	
I don’t know (never had a dental procedure)	2 (2.4)	2 (2.4)	
Have you taken pain-relieving medications after surgery?			0.016
No	25 (30.5)	32 (39.0)	
Yes	57 (69.5)	50 (61.0)	
First signs of post-operative pain were noticed			0.118
Less than 3 h after surgery	20 (24.4)	18 (22.0)	
3–6 h after surgery	38 (48.8)	48 (58.5)	
More than 6 h after surgery	22 (26.8)	16 (19.5)	
Please evaluate your real experience during Wide-Awake Local Anesthesia No Tourniquet surgery			<0.001
Experience was better than my expectations	50 (61.0)	60 (73.2)	
Experience matches my expectations	28 (34.1)	20 (24.4)	
Experience was worse than my expectations	4 (4.9)	2 (2.4)	
Are you suffering from persistent hand pain at the moment?			
No		68 (82.9)	
Yes		14 (17.1)	
Have you had any complications related to surgery?			
No		78 (95.1)	
Yes		4 (4.9)	
Are you satisfied with the outcome of performed surgery?			
No		16 (19.5)	
Yes		66 (80.5)	

**Table 3:** Persistent post-operative pain and complications in relation to patient evaluation of surgery experience.

Question	Evaluation Scores Median (Range)		*p* Value
	After 1 Month	After 6 Months	
Are you suffering from persistent hand pain at the moment?			
No			
Experience of pain during local anesthetic infiltration	4 (0–8)	3 (1–8)	<0.001
Experience of pain during surgery	1 (0–8)	1 (0–7)	0.002
Experience of pain after surgery	3 (0–9)	1 (0–8)	<0.001
Experience of anxiety before surgery	2.5 (0–10)	1 (0–10)	<0.001
Experience of anxiety during surgery	1.5 (0–10)	1 (0–10)	0.001
Experience of anxiety after surgery	0 (0–6)	0 (0–8)	0.207
Yes			
Experience of pain during local anesthetic infiltration	5 (2–8)	4 (2–5)	0.004
Experience of pain during surgery	2 (0–5)	2 (0–6)	0.670
Experience of pain after surgery	1 (0–8)	2 (0–5)	0.570
Experience of anxiety before surgery	0 (0–6)	1 (0–5)	0.670
Experience of anxiety during surgery	2 (0–7)	2 (0–5)	0.063
Experience of anxiety after surgery	0 (0–8)	0 (0–5)	0.414
Have you had any complications related to surgery?			
No			
Experience of pain during local anesthetic infiltration	4 (0–8)	3 (1–8)	<0.001
Experience of pain during surgery	1 (0–8)	1 (0–7)	0.004
Experience of pain after surgery	3 (0–9)	1 (0–8)	<0.001
Experience of anxiety before surgery	2 (0–10)	1 (0–6)	<0.001
Experience of anxiety during surgery	1 (0–10)	1 (0–10)	<0.001
Experience of anxiety after surgery	0 (0–8)	0 (0–8)	0.348
Yes			
Experience of pain during local anesthetic infiltration	8 (8–8)	7.5 (7–8)	0.157
Experience of pain during surgery	2.5 (0–5)	2.5 (0–5)	1.000
Experience of pain after surgery	5.5 (5–6)	4.5 (4–5)	0.157
Experience of anxiety before surgery	7.5 (5–10)	6 (2–10)	0.157
Experience of anxiety during surgery	5 (5–5)	5 (5–5)	1.000
Experience of anxiety after surgery	2.5 (0–5)	1 (0–2)	0.157

## Data Availability

The data presented in this study are available in article.
